# In vivo, intrinsic kinematics of the foot and ankle

**DOI:** 10.1186/1757-1146-5-S1-K5

**Published:** 2012-04-10

**Authors:** Toni Arndt, Chris Nester, Paul Lundgren, Arne Lundberg, Peter Wolf

**Affiliations:** 1 Karolinska Institute, Stockholm, 14186, Sweden; 2 The Swedish School of Sport and Health Sciences, Stockholm, 11486, Sweden; 3 University of Salford, Salford, M6 6PU, UK; 4 ETH Zurich, 8092, Switzerland

## Background

There are obvious problems involved in the accurate description of movement of the intrinsic bones within the foot and ankle. The 26 small bones are difficult, if not impossible to individually represent with standard skin mounted markers for motion analysis [1,2]. This international research collaboration has performed a number of studies in which invasively inserted intracortical pins are used for anchoring reflective markers, thereby providing a direct representation of the kinematics of the individual segments.

## Materials and methods

**Figure 1:**
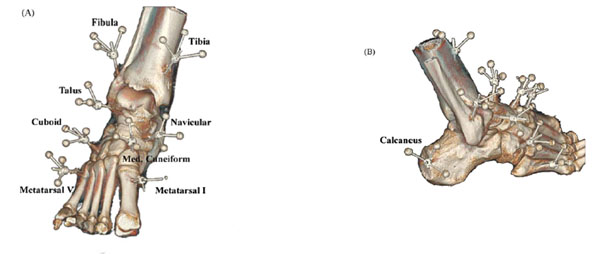
Computer tomography images of the marker locations on intracortical pins in foot and ankle segments.

A number of experimental sessions have been performed at Karolinska Institute. The intracortical pins were inserted by experienced orthopaedic surgeons under sterile conditions and using local anaesthetics (Figure 1). Triads of reflective markers were attached to the protruding ends of the pins and standard video based motion analysis (Qualysis, Sweden) conducted. Data have been published concerning walking [3] and slow running [4] and more recent work has for the first time investigated applied scientific questions such as the effect of shoe manipulations and in-shoe orthotics.

## Results

**Table 1:** Mean total ranges of motion (ROM) and standard deviations of motion about selected joints in the sagittal, frontal and transverse planes during walking. Data from six healthy, male subjects. From Lundgren et al., 2008.

plane	calc-tib		calc-tal		nav-tal		cub-calc		cub-nav	
	ROM [°]	SD	ROM [°]	SD	ROM [°]	SD	ROM [°]	SD	ROM [°]	SD

**sag**	17.0	2.1	6.8	1.4	8.4	1.1	9.7	5.2	7.2	2.4
**front**	11.3	3.5	9.8	1.8	14.9	6.1	11.3	3.9	8.8	4.4
**trans**	7.3	2.4	7.5	2.0	16.3	6.5	8.1	2.0	8.9	4.3

## Conclusions

A large range of fundamental data concerning foot and ankle kinematics during walking and running and with various manipulations have been collected and will be presented.
